# Morning Sickness of Pregnancy

**Published:** 1852-05

**Authors:** Seely Brownell

**Affiliations:** Chicago


ART. IV.—*Morning Sickness of Pregnancy.*

*Messrs. Editors*—I AM induced to make a few remarks upon this symptom of pregnancy, not that I intend to mention all of the grades of nausea, or to refer to every remedy advised by the numerous authors; for, though such remedies appear varied and numberless enough, yet so far as my experience goes they have done but little towards relieving the above named sickness. In 1849, I saw in the Boston Medical and Surgical Journal a recipe by E. Mallocks, M. D., which I have since used in two cases of the severest grade of the above symptom, as well as in a number of cases of less severity, in all of which relief was very decisive. The recipe to which I refer is bichloride of mercury, in half grain doses, in form of pill every morning before rising, until three doses have been taken, the patient observing at the sametime a proper diet, and keeping the bowels rather free with the usual laxatives. The favorable effect of the medicine in the above cases, though far from impressing me with a belief in its specific powers, has yet produced a strong desire to have it farther tested. SEELY BROWNELL, M.D.

CHICAGO, March 18, 1852.

